# Enhanced Electronic Properties of Pt@Ag Heterostructured Nanoparticles

**DOI:** 10.3390/s130607813

**Published:** 2013-06-18

**Authors:** Anh Thi Ngoc Dao, Derrick M. Mott, Koichi Higashimine, Shinya Maenosono

**Affiliations:** 1 Department of Materials Science, Japan Advanced Institute of Science and Technology, 1-1 Asahidai, Nomi, Ishikawa 923-1292, Japan; E-Mails: ngocanh@jaist.ac.jp (A.T.N.D.); shinya@jaist.ac.jp (S.M.); 2 Center for Nano Materials and Technology, Japan Advanced Institute of Science and Technology, 1-1 Asahidai, Nomi, Ishikawa 923-1292, Japan; E-Mail: koichi@jaist.ac.jp

**Keywords:** silver, platinum, nanoparticle, electronic transfer, core-shell, plasmonics, heterostructure

## Abstract

Platinum coated by silver nanoparticles was synthesized, which displays a unique structure where polycrystalline platinum particles are completely encapsulated in continuous monocrystalline silver shells. These particles display accentuated electronic properties, where the silver shells gain electron density from the platinum cores, imparting enhanced properties such as oxidation resistance. This electron transfer phenomenon is highly interfacial in nature, and the degree of electron transfer decreases as the thickness of silver shell increases. The nanoparticle structure and electronic properties are studied and the implication to creating sensing probes with enhanced robustness, sensitivity and controllable plasmonic properties is discussed.

## 1. Introduction

Plasmonic-based sensing probes consisting of nanoparticles (NP)s have become highly desirable because of their enhanced sensitivity, low cost, and easy to use nature [1,2]. Gold and silver are the most common type of metal studied for NP-based sensors because of their strong surface plasmon resonance (SPR) properties [3]. Silver is especially intriguing because it has the highest optical cross section for any metal, but still suffers from oxidation and an inability of its plasmonic properties to be tuned for a desired application [4]. As a result, NPs with complex structures (heterostructures) such as core@shell NPs have the potential to display the strong optical properties of silver, be robust and possess controllable plasmonic properties [5]. Several silver-based heterostructured NP systems have been created in an attempt to realize robust and active NP sensing probes, however silver NPs remain sensitive to oxidation, or display compromised plasmonic properties [4,6,7]. For example, Ag@Au or AuAg alloy NPs have proven limited in exhibiting the same level of plasmonic activity as pure silver NPs, and oftentimes the structure of these particles is challenging to control precisely [6–8]. Instead, a new approach to the creation of silver based heterostructured NPs is required to more fully understand the relationship between NP structure and the resulting optical/plasmonic properties. Recently, it was shown that by understanding the electronic characteristics of silver at the nanoscale, insight can be gained into how to create heterostructured silver based sensing probes with desired and controllable properties [9,10]. Specifically, it was shown that Au@Ag NPs display a unique electron transfer phenomenon that results in the silver shell becoming oxidation resistant while retaining its strong plasmonic properties [9,11]. By extending this phenomenon to other silver based NP systems, insight can be gained into how to manipulate the particle structure and composition towards the desired characteristics. With this goal in mind, we created a series of different sized platinum particles and coated them in silver shells of various thicknesses. In this case, platinum was chosen as a core material because of its status as a noble metal, its fcc crystal structure (the same as for silver), and its chemical similarity to gold. The resulting particles were characterized in terms of their structural/composition properties, and then the electronic properties of these probes were analyzed by using X-Ray Photoelectron Spectroscopy. The results demonstrate that the electronic transfer phenomenon can be extended to a wide range of heterostructure systems, and provides insight into how to exploit electronic transfer to create silver based sensing probes with enhanced robustness, high optical/plasmonic activity and plasmonic characteristics that can be tuned for a desired application.

## 2. Experimental

### 2.1. Chemicals

Chloroplatinic acid solution (H_2_PtCl_6_) 8% by weight, sodium citrate tribasic dihydrate (Na_3_C_6_H_5_O_7_.2H_2_O) 99%, citric acid (C_6_H_8_O_7_) 99.5%, and sodium borohydride (NaBH_4_) 98% were obtained from Sigma-Aldrich (Kanazawa, Japan). L-ascorbic acid (C_6_H_8_O_6_) 99.5% was obtained from Wako Pure Chemical Industries (Osaka, Japan). Water was purified with a Millipore Direct-Q system (18.2 MΩ).

### 2.2. Nanoparticle Synthesis

#### 2.2.1. Synthesis of Platinum NPs

A platinum suspension was prepared in aqueous solution following a multistep seed-mediated growth procedure by Bigall *et al.* [12]. Initially, small platinum seeds of 4.5 ± 0.7 nm in diameter were prepared. First, an 8% (by weight) chloroplatinic acid solution (0.086 mL) was added to boiling purified water (43.314 mL). After 1 minute, a solution (1.1 mL) containing 1% sodium citrate and 0.05% citric acid was added, followed half a minute later by a quick injection of a freshly prepared 0.08% sodium borohydride solution (5.5 mL) also containing 1% sodium citrate and 0.05% citric acid. After 10 minutes, the sol solution was cooled down to room temperature. The platinum seeds obtained here were used in preparing larger sized platinum NPs in further reaction steps [12].

Larger sized platinum NPs were prepared by adding the platinum seed solution (1 mL) to purified water (28.12 mL), 8% chloroplatinic acid solution, 25% citric acid solution (0.35 mL), and then 1.25% L-ascorbic acid solution (0.5 mL) containing 1% sodium citrate. The mixture was slowly heated to the boiling point and left to react for 30 minutes with stirring, and then cooled down to room temperature. The amount of chloroplatinic acid solution used in various reactions included 0.017, 0.03, 0.045 and 0.334 mL to obtain 25.7 ± 4.3, 31.3 ± 5.1, 39.8 ± 4.6 and 99.3 ± 3.7 nm platinum NPs, respectively. The reaction products collected by centrifugation (centrifuge operated at 3,000–8,500 rpm depending on nanoparticle size) were washed three times with 0.5% sodium citrate solution, and then stored by dispersing in 0.5% sodium citrate solution.

#### 2.2.2. Synthesis of Pt@Ag NPs

The as-synthesized citrate-capped platinum NPs were used as core particles in the preparation of Pt@Ag core@shell NPs [9]. The platinum NP dispersion (20 mL) was brought to reflux with stirring until boiling, and then aqueous solution of AgNO_3_ (5 mL) and sodium citrate (5 mL, 0.0086%) were simultaneously added dropwise. The reaction solution was refluxed for 20 minutes and then left to cool to room temperature. The silver shell thickness of Pt@Ag core@shell NPs was controlled by increasing the concentration of AgNO_3_ added in the reaction. To 25.7 nm platinum NPs, an 8.1 nm Ag shell thickness was deposited by adding a 1.18 mM solution of AgNO_3_. To 39.8 nm platinum NPs, AgNO_3_ concentrations including 0.17, 0.58, and 2.19 mM were used to prepare Ag shell thicknesses of 1.3, 5.2, and 9.1 nm, respectively.

#### 2.2.3. Synthesis of Silver NPs

Silver NPs were synthesized to compare the electronic characteristic with silver in the shell of Pt@Ag core@shell NPs [13]. Briefly, a solution of AgNO_3_ (45 mL, 1 mM) was stirred in a 100 mL round bottom flask and purged with argon gas for 15 minutes. This solution was heated to reflux at 100 °C, and then a 33.7 mM sodium citrate solution (5 mL) was added. After 5 minutes of boiling, the color of the solution turned to yellow, and after 15 minutes it became opaque. The reaction solution was cooled to room temperature after 1 hour refluxing, and then the opaque dispersion was centrifuged at 5,000 rpm for 10 minutes. After the centrifugation, the upper part of the solution became a transparent light-yellow color. This part contained the final Ag NPs with the average sizes of 35.0 nm and was taken to a container for further experiments.

### 2.3. Instrumentation and Measurements

Synthesized NPs were characterized by transmission electron microscopy (TEM), High Resolution TEM (HRTEM), scanning TEM equipped with a high angle annular dark-field detector (STEM-HAADF), energy dispersive X-ray spectroscopy (EDS), X-ray photoelectron spectroscopy (XPS), X-ray diffraction (XRD) and ultraviolet-visible spectroscopy (UV-Vis). TEM observations were performed on a Hitachi H-7650 instrument operated at 100 kV. STEM-HAADF and EDS elemental mapping were performed on a JEOL JEM-ARM200F operated at 200 kV, the nominal resolution is 0.8 Å. Samples for TEM, EDS and STEM-HAADF were prepared by dropping the suspended NPs onto a carbon-coated copper grid and drying in air overnight. XPS analysis was carried out on a Shimadzu Kratos AXIS-ULTRA DLD high performance XPS system. Photoelectrons were excited by monochromated Al K_α_ radiation. Detection was done with a delay-line detector (DLD) and a concentric hemispherical analyzer (CHA). The X-ray tube was operated at 150 W. The pass energy of the CHA was 20 eV for narrow-scan spectra. The analyzed area on the specimen surface was 300 × 700 μm^2^ and was located in the center of the irradiated region. For the XPS sample preparation, the precipitated NPs were deposited on a molybdenum substrate and dried in vacuum. The instrument was operated at a vacuum level of 1 × 10^−8^ Torr. XRD patterns were collected in reflection geometry using a Rigaku SmartLab X-ray diffractometer at room temperature with Cu K_α_ radiation (wavelength 1.542 Å, 40 kV, 30 mA). UV-Vis spectra were collected in the range of 300 to 1,100 nm using a Perkin-Elmer Lambda 35 UV-Vis spectrometer.

## 3. Results and Discussion

The results and discussion section is split into four main parts. First we study the general NP characteristics such as size, shape, composition and structural parameters. This section is followed by analysis of the particle crystalline properties of the Pt@Ag NPs. Next we address the electronic properties in an in-depth XPS study, probing the effect of increasing thickness of silver on the platinum particle surface and the impact on the NP electronic structure. Finally, we comment on the implications of the electronic transfer effect on creating sensitive and robust NP based sensing probes. Throughout the results and discussion section, the notation Pt_x_@Ag_y_ will be used where x and y represent the mean platinum core size or silver shell thickness, respectively. As will be discussed, a wide range of particles with various core size and shell thickness were created, leading to a large amount of redundant data. As a result, we often present a representative sample analysis in the interest of creating a cohesive study.

### 3.1. General Morphology and Optical Properties

**Figure 1.**
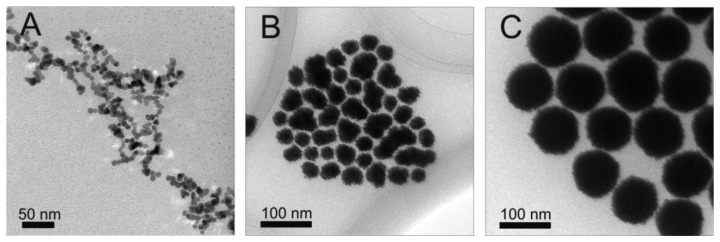
TEM images of platinum nanoparticles with a size of 4.5 (**A**); 31.3 (**B**); and 99.3 (**C**) nm.

Initially, platinum core NPs were synthesized. These particles serve as the basis for creating Pt@Ag NPs in further studies, and their size, structure and morphology play a key role in the characteristics of the final Pt@Ag NPs. Figure 1 shows a set of representative TEM images collected for platinum particles synthesized with increasing size. First, following the above synthetic protocol, relatively small platinum seed particles were created. These seed NPs have a size of 4.5 ± 0.7 nm and are roughly spherical in shape (though they do appear to be slightly clustered). The seed NPs were used in subsequent reaction steps to produce incrementally larger platinum NPs with average sizes of 25.7 ± 4.3, 31.3 ± 5.1, 39.8 ± 4.6 and 99.3 ± 3.7 nm. In general, the platinum NP size and morphology became visually more uniform, but display highly roughened surfaces. The high degree of surface roughness may suggest that the NPs are polycrystalline in nature. The NP sizes were determined using TEM analysis, however in light of the fact that the smaller platinum NPs are slightly clustered, and the larger particles are polycrystalline in nature with highly roughened surfaces, the particle size distributions have a limited amount of statistical significance because of the uncertainty associated in judging the particle sizes graphically. Note that while different ratios of precursor were used (to obtain different particle sizes) the average sizes of platinum particle created in this work are consistent with those created previously [12].

**Figure 2.**
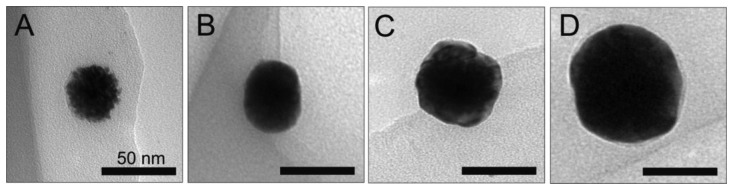
TEM images of a platinum core particle (39.8 nm) (**A**) and Pt@Ag particles with an optimal silver shell thickness of 1.3 (**B**); 5.2 (**C**); and 9.1 (**D**) nm.

Next, the platinum NPs were used as cores for the deposition of silver on the particle surface. Figure 2 shows the TEM images collected for a single bare platinum core with a size of 39.8 nm, as well as particles with incrementally increasing silver shell thicknesses of 1.3, 5.2 and 9.1 nm. Immediately after the initial coating with silver, the particle surface becomes much smoother in appearance. This trend continues for increasing silver shell thicknesses.

**Figure 3.**
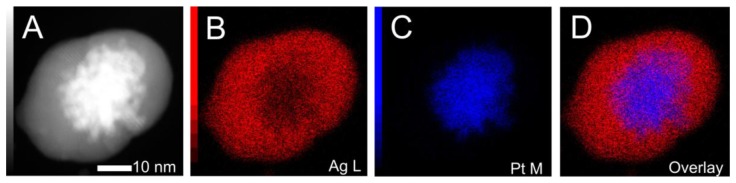
STEM-HAADF image (**A**) and the corresponding EDS elemental maps for silver L line (**B**); platinum M line (**C**); and an overlay of the two maps (**D**) for a single Pt_25.7_@Ag_8.1_ NP.

After coating the platinum particles in a silver shell, the original platinum cores cannot be directly observed in traditional TEM analysis. In order to elucidate the core@shell structural nature of the NPs, we performed STEM-HAADF analysis coupled with EDS elemental mapping technique. The combination of these two analysis techniques gives an excellent 2-dimensional structural picture of the created materials. Figure 3 shows the STEM-HAADF image of a single Pt_25.7_@Ag_8.1_ NP as well as EDS elemental maps taken of the same particle for silver L line, platinum M line and an overlay of the two maps. First, the STEM-HAADF image clearly shows that the platinum particle exists in the center of the newly formed Pt_25.7_@Ag_8.1_ NP. This is indicated by the very bright core region observed in the image surrounded by a less bright peripheral area. The STEM-HAADF technique offers enhanced contrast sensitivity to the elements atomic number, essentially larger atomic number elements appear relatively more bright in the image, known as Z contrast [14]. The mapping images for platinum and silver show the relative location of these two elements, with the overlay map clearly demonstrating that platinum is confined to the core region of the particle while silver surrounds the platinum core. The data provides good evidence of the core@shell structural nature of these Pt@Ag NPs.

**Figure 4.**
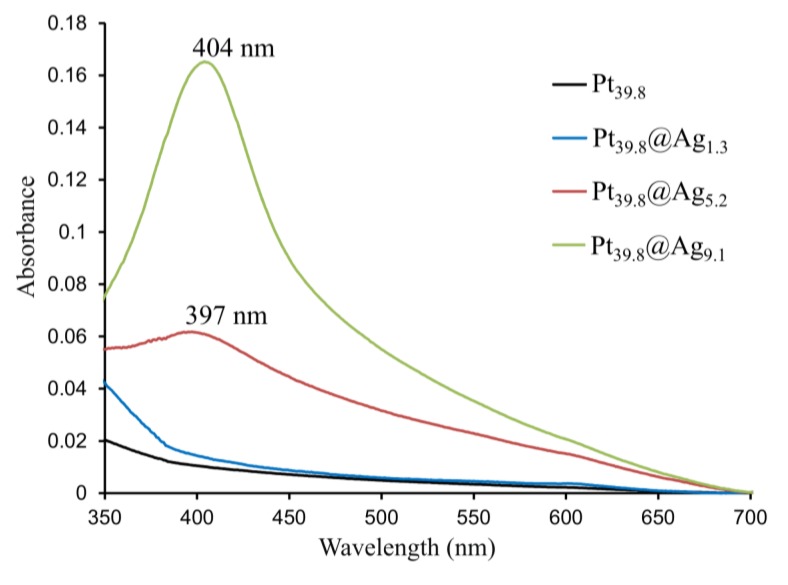
UV-Vis spectra for platinum core particle (39.8 nm) and Pt_39.8_@Ag_y_ particles with an optimal silver shell thickness of 1.4; 5.2; and 9.1 nm.

The bare platinum and Pt@Ag NPs display plasmonic properties which were studied using UV-Vis spectroscopy. Figure 4 shows the UV-Vis spectra taken for four different types of NP including bare platinum, Pt_39.8_@Ag_1.3_, Pt_39.8_@Ag_5.2_ and Pt_39.8_@Ag_9.1_ NPs.

It can be observed that the bare platinum and Pt_39.8_@Ag_1.3_ samples do not display surface plasmon resonance (SPR) in the visible region, but the Pt_39.8_@Ag_5.2_ and Pt_39.8_@Ag_9.1_ exhibit a clear SPR band around 400 nm, growing in intensity as the silver shell thickness increases. The position of this SPR band is typical for silver NPs [4,9,10].

### 3.2. Unique Crystalline Structure

**Figure 5.**
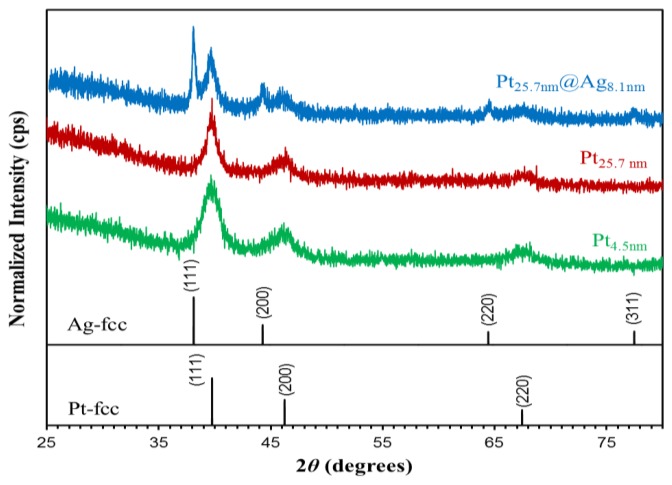
XRD pattern of platinum seeds (4.5 nm), platinum NPs (25.7 nm) and Pt_25.7_@Ag_8.1_ NPs. The reference peak positions for fcc phase platinum and silver are also shown.

The larger sized platinum NPs as well as the Pt@Ag structures possess a unique crystal structure as evidenced by XRD analysis. Figure 5 shows the XRD patterns of three NP samples including platinum NPs with a size of both 4.5 and 25.7 nm, as well as for Pt_25.7_@Ag_8.1_ NPs.

The graph also includes the location of reference peaks for fcc silver and platinum [15,16]. For both sizes of platinum NP, the pattern shows broad peaks at locations consistent for fcc phase platinum. After coating with silver, peaks originating from the platinum metal are still observed with new sharper peaks appearing at the location for fcc phase silver. Scherrer analysis of the primary 111 peaks for both platinum and silver was used to study the crystalline size properties of the particles and revealed a mean crystalline size of 5.1 nm for smaller platinum NPs, 7.3 nm for larger platinum NPs, and 20.2 nm for the silver shells deposited onto the larger sized platinum cores. In this case, the silver has a larger mean crystalline size than the relative shell thickness because the shell is continuous and encapsulates the entire core platinum particle, reflecting the monocrystalline nature of the silver shell. The results reveal a unique crystalline structure for this particle system where larger sized platinum NPs are polycrystalline in nature, while the deposited silver shell is monocrystalline in nature as evidenced by the large mean silver crystal size measured using Scherrer analysis. The resulting morphological structure is a cluster of smaller platinum crystals encapsulated in a continuous silver casing, which could influence the resulting electronic properties to a large degree. Equation (1) shows the Scherrer equation used where *d* (nm) is the mean size of the crystalline domain (crystalline size) of the NPs, *K* is the shape factor (dimensionless) with a value of 0.9 (but depends on the shape of NPs), *λ* [nm] is the X-ray wavelength (*λ* = 0.15418 nm), *β* (rad) is the peak width at half the maximum intensity (FWHM) and *θ* [rad] is the peak position (Bragg angle) [17]. Table 1 lists the determined parameters for the 111 peaks for both platinum and silver metals, as well as the derived mean crystalline size.

(1) $$d=\frac{K\lambda }{\beta \cos\theta }$$

**Figure 6.**
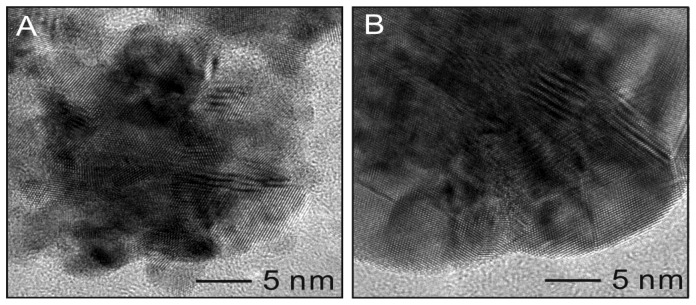
HRTEM images of a bare platinum (25.7 nm) NP (**A**) and for a Pt_25.7_@Ag_8.1_ NP (**B**).

HRTEM analysis provides additional confirmation of the crystalline structure of the Pt@Ag NPs. Figure 6 shows HRTEM images of a single platinum NP (25.7 nm) as well as for a single Pt_25.7_@Ag_8.1_ NP. The bare platinum NP shows a clustered morphology with atomic lattice planes facing several different directions, suggesting a polycrystalline nature. Measurement of the atomic plane spacing gives an average value of 0.220 nm, consistent with the 111 plane of fcc platinum [15]. For the silver coated sample, the outermost area appears more continuous in terms of the crystal lattice. The measurement of the atomic plane spacing at the periphery of the particle gives a value of 0.249 nm, consistent with the 111 plane of fcc silver [16].

### 3.3. Electronic Structure

In order to gain a diagnostic assessment of the electronic properties of the silver shell in Pt@Ag NPs, XPS analysis was performed on a series of Pt@Ag NPs with increasing shell thickness. A total of five samples were analyzed including pure silver (35.0 nm) NPs, Pt_39.8_@Ag_1.3_, Pt_39.8_@Ag_5.2_, Pt_39.8_@Ag_9.1_ and pure platinum NPs (39.8 nm). Figure 7 shows the collected XPS spectra in the Ag 3d area while Figure 8 shows the XPS spectra collected in the Pt 4f region.

Both areas show single phase peaks with no signs of oxidation for each sample. Note that the silver NPs also do not exhibit a characteristic oxide peak (such as that formed as a silver oxide layer on the silver NP surface) because the particles were synthesized under an inert atmosphere and were quickly transferred for the analysis, avoiding the characteristic oxidation. This simplifies the analysis of the electronic transfer by eliminating the overlapping oxide peak in the XPS spectra. Precise peak parameters were determined by using a Gaussian-Lorentzian (G-L) mixed function to fit the experimental data [18]. The inserts to each graph in Figure 7 and 8 show the experimentally collected data (solid lines) as well as the fitted data using the G-L mixed function (dotted lines). Equation (2) describes the G-L mixed function while the derived peak parameters for Ag 3d area and Pt 4f area spectra are listed in Table 2 and Table 3, respectively.

The Gaussian-Lorentzian mixed function was used to analyze the Ag 3d peak shape [18]:

(2) $$f(x)=\frac{l_{0}}{\left\{1+M{\left(x-x_{0}\right)}^{2}/\Gamma^{2}\right\}\exp\left\{(1-M)\ln2{\left(x-x_{0}\right)}^{2}/\Gamma^{2}\right\}}$$

where *l_0_*, *x_0_*, *x*, *Γ* and *M* are the peak height, the peak BE, the BE, a parameter for the peak width, and the G-L mixing ratio, respectively. To introduce the asymmetry into the G-L mixed function, the following variable transformation is incorporated in Equation (2) [18].

(3) $$(x-x_{0})\to \frac{\left(x-x_{0}\right)}{\left\{1+\alpha (x-x_{0})/\Gamma \right\}}$$

**Figure 9.**
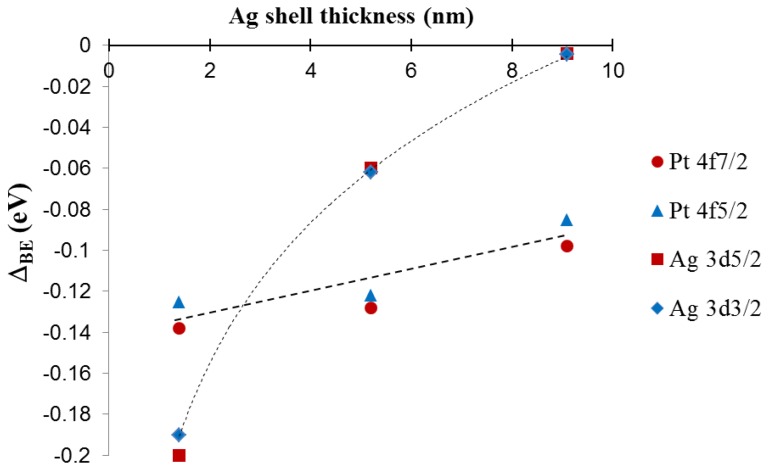
The Pt 4f and Ag 3d peak energy shift plotted as a function of silver shell thickness for Pt_39.8_@Ag*_y_* (*y* = 1.3, 5.2, and 9.1 nm) NPs. The peak energy shift Δ_BE_ = BE_core-shell_ − BE_Pt_ (or BE_Ag_).

For silver, there is a clear negative shift in the 3d peak energy for Pt_39.8_@Ag_y_ samples as compared to pure silver NPs. The most pronounced shift is observed for the thinnest silver shell with peak energy moving back towards the value for pure silver as the silver shell thickness increases. The platinum 4f peak position also shows a subtle trend in the data with a negative energy shift in the peak position as compared to pure platinum NPs, but in this case as the silver shell thickness increases, the platinum peak positions move slightly towards the expected position for pure platinum. The trends in the XPS spectra peak positions are summarized in Figure 9, which shows a plot of peak energy shift in silver 3d and platinum 4f peaks versus the silver shell thickness.

The lines connecting the data points in the graph are included to assist in visual assessment of the relative data point positions. The observations are broadly consistent with an electron transfer phenomenon where the silver shell gains electron density from the core metal, in this case platinum [9,11,19]. The phenomenon is more pronounced for thinner silver shells because of the interfacial nature of the electron transfer and ultimately results in enhancement of the chemical properties of the silver shell. For instance, an electron rich silver shell is expected to become resistant to oxidation [9,10,19]. A simplified way to understand the observed shift in the XPS peaks is through the effect of relative electron density on the resulting binding energy of peaks in the XPS spectra. As silver gains electron density, it becomes relatively easier to remove electrons from silver sites, hence the binding energy is negatively shifted. For platinum, the 4f peak is also negatively shifted, which is counter intuitive, however this arises from the fact that the electronic transfer process is complex in nature. In reality, the true electronic configuration of the Pt@Ag NP structure may consist of electron donation and back-donation in various orbitals of the metals, which may account for the observation of a concurrent negative shift in silver 3d and platinum 4f orbitals. In addition, because platinum core NPs actually consist of small platinum nanocrystals and have highly roughened surfaces while Pt@Ag NPs have more smooth surfaces with relatively large silver grain sizes, there should be some differences in initial and final state effects of the electron emission process. This may be one of the reasons why the negative shift in platinum 4f peaks is observed. The electronic transfer phenomenon is supported by the observation of a sharp decrease in the BE shift for silver as the silver shell thickness increases, while for platinum the shift in binding energy only changes by a small degree. This shows that the electronic enrichment of silver in the 3d orbital is interfacial in nature, which can only arise in the electronic transfer process. A study on the electronic configuration of bare platinum versus coated NPs using X-ray Absorption Near Edge Structure (XANES) analysis could provide additional information on the electronic configuration of these materials [11].

### 3.4. Implication of the Electronic Properties to Sensing Characteristics

**Scheme 1.**
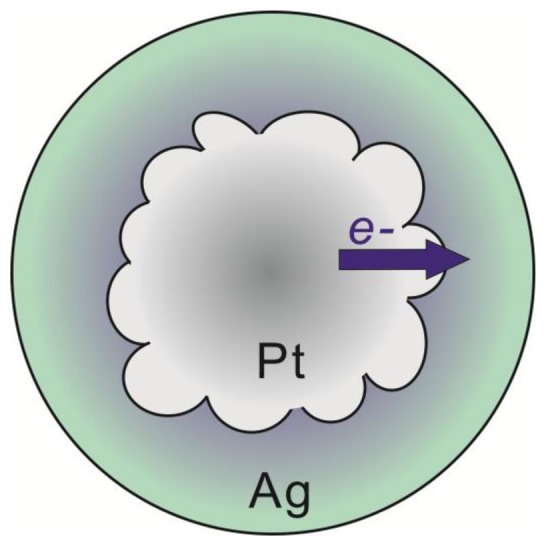
Illustration of the particle structure and general electron transfer process. The purple color at the interface of platinum and silver represents the interfacial nature of the electron transfer.

The unique electronic properties displayed by the Pt@Ag NP system show that NP heterostructures can lead to materials with controllable and enhanced properties beyond what is offered by monometallic or alloyed NP systems. By controlling the structural parameters of these NP heterostructures, new or enhanced characteristics can be extracted such as resistance to oxidation, robustness, or maximizing plasmonic properties. Such ability is highly advantageous for use in sensing probe systems where these characteristics are critical for creating highly sensitive probes. In the Pt@Ag NP case, by avoiding the oxidation of silver, bio-molecular sensing probes can be formed which are reliable and highly sensitive because the probes do not undergo oxidative degradation. In addition, by understanding the electron transfer mechanism, the plasmonic properties of this class of core@shell structure can be further tailored for desired applications allowing sensing probes to be developed that possess higher sensitivity and accuracy [19]. In general, the electronic transfer can be understood as the enrichment of electron density at silver sites at the expense of electron density at platinum sites in the core@shell structure. The electronic characteristics are analogous to what would occur in an alloy of platinum and silver, yet the nanostructures here exhibit clear phase segregation. As a result of the small Pt@Ag NP size, alloy-like electronic properties emerge at the interface of the two metals, which in this case is likely enhanced by the polycrystalline nature of the platinum NP core surface. The highly roughened surface of the platinum NPs creates a larger surface area at the interface of platinum and silver in the core@shell structure. Because the electronic transfer phenomenon is interfacial in nature, this provides more sites for the electron transfer, leading to a relatively higher degree of electron enrichment for the silver shell. This observation is especially apparent when comparing the degree of electron transfer in the case of Au@Ag NPs [9] and for the Pt@Ag particles created in this work. For 14.4 nm gold particles coated in a silver shell with a thickness of 1.0 nm, a negative shift of 0.1 eV in the Ag 3d peak in the XPS spectrum is observed, while for the Pt_39.8_Ag_1.3_ particles created here, a shift of nearly 0.2 eV is observed. While the core particle sizes in these two systems are different, the observation of accentuated electronic transfer in the case of using a platinum core in the Pt@Ag NP structure illustrates the potential for this system to express unique and enhanced sensing characteristics as a result of the strengthened electronic transfer. Scheme 1 represents the general structure and processes for electron transfer occurring in the Pt@Ag NP system, including representation of the interfacial nature of the phenomenon shown by the color gradient in the silver shell.

## 4. Conclusions and Future Outlook

A series of platinum coated by silver (Pt@Ag) NPs have been synthesized which display a well-defined structure where polycrystalline platinum particles are completely encapsulated in a continuous monocrystalline silver shell. The understanding of the electron transfer phenomenon in the platinum-silver NP system enhances the ability to purposely manipulate the stability and plasmonic properties of core@shell NP sensing probes and provides fundamental insight into the electronic characteristics of nano-heterostructures. The Pt@Ag particles display surface plasmon resonance properties which could be exploited in plasmonic sensing probe applications. More significantly, these NPs display unique electronic properties where the silver shell gains electron density from the platinum cores, as revealed by analysis of the XPS spectra of these materials. These unique electronic properties give insight into how this class of heterostructured NP can be manipulated to create new and powerful sensing probes with enhanced properties such as oxidation resistance, robustness, plasmonic activity and other properties.

## Figures and Tables

**Figure 8.**
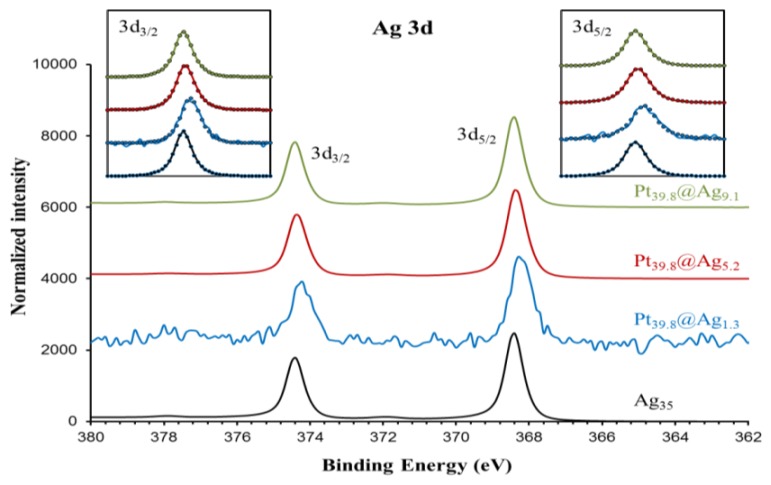
XPS spectra for platinum core particle (39.8 nm) and Pt_39.8_@Ag_y_ particles with an optimal silver shell thickness of 1.3; 5.2; and 9.1 nm. The inserted graphs show the Pt 4f_7/2_ and 4f_5/2_ peaks (solid curves) and the fitting with the asymmetric G-L mixed function (circles).

**Figure 8.**
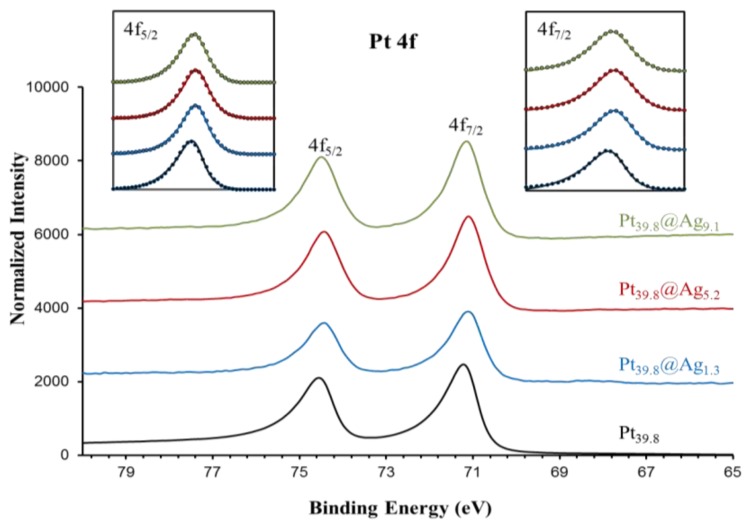
XPS spectra for platinum core particle (39.8 nm) (**A**) and Pt_39.8_@Ag_y_ particles with an optimal silver shell thickness of 1.3 (**B**); 5.2 (**C**); and 9.1 (**D**) nm. The inserted graphs show the Pt 4f_7/2_ and 4f_5/2_ peaks (solid curves) and the fitting with the asymmetric G-L mixed function (circles).

**Table 1.** Peak parameters and mean crystalline size derived from Scherrer analysis of the 111 peaks in XRD patters.

		**2*θ* (degrees)**	***β* (degrees)**	***d* (nm)**
Pt_4.5nm_		39.76	1.73	5.1
Pt_25.7nm_		39.64	1.20	7.3
Pt_25.7nm_@Ag_8.1nm_	Pt	39.48	1.21	7.3
	Ag	37.94	0.43	20.2

**Table 2.** Peak parameters obtained by curve fit of Ag 3d peaks using a G-L mixed function.

		**Pt_39.8_@Ag_1.3_**	**Pt_39.8_@Ag_5.2_**	**Pt_39.8_@Ag_9.1_**	**Ag_35.0_**
3d_5/2_	Peak position	368.21	368.35	368.41	368.41
	FWHM	0.33	0.31	0.31	0.29
	M	0.79	0.87	0.84	0.90
	α	0	0	0	0

3d_3/2_	Peak position	374.22	374.35	374.41	374.41
	FWHM	0.33	0.30	0.29	0.29
	M	0.94	0.85	0.84	0.83
	α	0	0	0	0

Spin-orbit intensity ratio		1.48	1.46	1.43	1.52

**Table 3.** Peak parameters obtained by curve fit of Pt 4f peaks using a G-L mixed function.

		**Pt_39.8_@Ag_1.3_**	**Pt_39.8_@Ag_5.2_**	**Pt_39.8_@Ag_9.1_**	**Pt_39.8_**
4f_7/2_	Peak position	71.09	71.10	71.13	71.23
	FWHM	0.46	0.46	0.47	0.46
	M	0.73	0.74	0.70	0.73
	α	0.09	0.06	0.09	0.10

4f_5/2_	Peak position	74.43	74.43	74.47	74.56
	FWHM	0.45	0.47	0.47	0.44
	M	0.61	0.67	0.62	0.63

	α	0.12	0.08	0.09	0.13

Spin-orbit intensity ratio		1.36	1.34	1.35	1.39
